# Differential metabolites and key pathways in quinoa rhizosphere responding to salt stress: implications for salt tolerance

**DOI:** 10.1186/s12870-026-09698-x

**Published:** 2026-08-08

**Authors:** Jiaqi Gu, Yuanru Yang, Haiying Guo, Yiran Zhao, Yu Chun, Sarina Bao

**Affiliations:** 1 College of Life Science and Technology, Inner Mongolia Normal University, Hohhot, 010020 China; 2 Agricultural and Animal Husbandry Technology Promotion Center of Chahar Right Middle Banner, Wul-anchabu City, 013550 China

**Keywords:** Quinoa, Salt stress, Rhizosphere metabolites, Metabolome

## Abstract

**Background:**

As an important salt-alkali-tolerant crop, quinoa can respond to salt stress through various pathways. However, the response mechanisms of the rhizosphere microenvironment to salt stress require further exploration. This study aimed to investigate the effects of salt stress on the metabolic profile of quinoa rhizosphere soil and to explore its salt tolerance mechanisms at the metabolic level.

**Results:**

Using Ultra-high performance liquid chromatography coupled with quadrupole-Orbitrap mass spectrometry (UHPLC-Q-Orbitrap-MS)based untargeted metabolomics, combined with multivariate statistical analysis and KEGG pathway enrichment analysis, this study systematically compared the metabolic differences in rhizosphere soil between the salt-stressed group and the control group. The results showed that a total of 856 metabolites were identified, with 439 significantly differential metabolites screened out, including 344 up-regulated and 95 down-regulated metabolites. The differential metabolites primarily included lipids and lipid-like molecules, organic acids and their derivatives, organooxygen compounds, organic nitrogen compounds, and heterocyclic compounds. The accumulation of key differential metabolites indicated that quinoa responds to salt stress through multiple synergistic strategies, such as osmotic regulation, antioxidant defense, membrane lipid remodeling, as well as hormone signaling and acetic acid metabolism. KEGG enrichment analysis further revealed that pathways including phenylalanine metabolism, amino acid biosynthesis, and ATP-Binding Cassette transporters (ABC transporters) were significantly enriched, forming a systemic adaptation network encompassing defense substance synthesis, osmotic protection deployment, and substance transport regulation.

**Conclusions:**

In conclusion, composite saline-alkali stress induces extensive reprogramming of metabolite composition in quinoa rhizosphere soil. The observed shifts in rhizosphere metabolites are closely linked to the salt tolerance adaptation of quinoa. This study offers a theoretical foundation for understanding rhizosphere metabolic responses and may inform strategies for improving crop performance in saline-alkali lands.

## Background

Quinoa (*Chenopodium quinoa Wild*), an Amaranthaceae crop originating from the Andean region, is widely recognized as a ‘superfood’ with significant development potential and a crucial crop for ensuring future food security. This recognition stems from its seeds being rich in nutritional components such as protein, essential amino acids, minerals, dietary fiber, and vitamins, coupled with its characteristics of low glycemic index and gluten-free properties. Beyond its utilization as a staple food, both the seedlings and tender leaves of quinoa are also edible, which further enhances its overall value [1]. Notably, owing to its exceptional salt and alkali tolerance, quinoa has emerged as an ideal crop for the improvement of saline-alkali soils.

The salt tolerance of quinoa is not mediated by a single mechanism but rather represents a multi-level, integrated adaptive strategy involving multiple pathways. As systematically summarized by Qura Tul Ain et al. [2], these mechanisms encompass: allelic variation and rapid induction of stress-responsive genes at the genetic level; osmotic and ion homeostasis at the physiological level; activation of the antioxidant defense system at the biochemical level; and adjustments in root system architecture at the morphological level.

Soil salinization stands as one of the primary environmental stress factors constraining global agricultural production, affecting approximately 20% of irrigated farmland and leading to decreased soil productivity, inhibited crop growth, and even abandonment of cultivated land [3]. China possesses approximately 99.13 million hectares of salt-alkali land, with the area continuing to expand, posing a serious threat to the nation’s food security [4]. Elucidating the salt tolerance mechanisms of quinoa holds great significance in this context.

The rhizosphere refers to the microzone where plant roots directly interact with soil microorganisms. As one of the most biologically active interfaces on Earth, it profoundly regulates nutrient acquisition, stress tolerance and overall productivity of plants [5]. Root exudates constitute the primary component of plant rhizosphere deposition and serve as the core factor driving plant-soil chemical interactions. Through regulating soil biochemical metabolic pathways, they profoundly influence plant stress tolerance [6]. In recent years, studies have gradually revealed the pivotal role of the rhizosphere metabolome in plant response to salt stress. Shi et al. [7] discovered that in intercropping systems, the rhizospheres of salt-tolerant sorghum and peanut exhibited distinct metabolite profiles, which facilitated salt stress tolerance by modulating the host’s energy metabolic pathways. Subsequent research by Mohammad et al. [8] demonstrated that this intercropping advantage was partially attributable to the regulation of soil sugar metabolic pathways, which consequently altered the rhizosphere metabolic environment. Similarly, a study conducted by Yinxiao W et al. [9] on rice indicated that overexpression of the salt-tolerant transcription factor Oryza sativa DREB-related AP2 1 (OsDRAP1) mediated salt tolerance phenotypes through the remodeling of amino acid and carbohydrate metabolic pathways within the rhizosphere. Research by Chen et al. [10]demonstrated that 13 amino acid-related metabolic pathways in Tamarix roots participate in salt stress resistance, with increased proline content and α-ketobutyrate production representing effective salt tolerance mechanisms. These findings collectively indicate a core mechanism: under salt stress conditions, plants release root exudates that modify the biochemical state of the rhizosphere environment (including the composition of organic acids, sugars, and amino acids), thereby indirectly facilitating the maintenance of ion balance, energy supply, and redox homeostasis.

Significant variations exist in the capacity of plants to regulate the rhizosphere chemical microenvironment under salt stress. Studies by Li et al. [11] revealed that under salt stress conditions, plant rhizosphere secretions differ substantially in their metabolite composition, which may be the determining factor for variations in salt tolerance. Moreover, salt stress directly interferes with the internal hormone signaling networks of plants. Research conducted by Smolko et al. [12] in Arabidopsis revealed that short-term salt stress induces drastic changes in auxin content and the redistribution of its signaling from the root tip quiescent center to the elongation zone. This hormonal reprogramming inevitably influences the composition and flow of root exudates.

Most existing multi-omics studies on salt tolerance of quinoa only adopt single NaCl treatment to simulate salt stress, rather than combined NaCl-NaHCO₃ saline-alkali conditions. In addition, their detection materials are mainly leaves and whole seedlings above ground, lacking systematic analysis of the rhizosphere microenvironment. Imran et al. [13] revealed that various quinoa genotypes cope with single salt stress via the USP gene network and osmotic metabolism pathways through multi-omics integrated analysis. Li and Zhang [14] illustrated the time-series responses of antioxidant and photosynthetic pathways in quinoa seedlings under short-term NaCl stress based on time-course transcriptome data. However, neither of the two studies investigated rhizosphere metabolism. Meanwhile, most research on the synergistic salt-resistance interactions between root exudates and microorganisms takes fruit trees as research objects. Yang et al. [15] integrated rhizosphere bacterial community profiling with root exudate metabolomics and clarified that Pyrus betulifolia recruits beneficial bacteria through specific root exudates to improve saline-alkali tolerance, yet such interaction mechanisms have not been explored in quinoa. Taken together, systematic research focusing on quinoa rhizosphere metabolism under composite saline-alkali stress is still absent, and the regulatory mechanism of saline-alkali tolerance mediated by quinoa root exudates remains unclear. In this study, NaCl-NaHCO₃ mixed solution was used to mimic natural saline-alkali habitats, and the rhizosphere metabolic response characteristics of quinoa were analyzed, aiming to supplement the theoretical basis for deciphering quinoa salt tolerance and saline-alkali land utilization.

## Methods

The pot experiment using the quinoa variety “Qingli 2” was conducted in the Plant Physiology Laboratory of the College of Life Science and Technology at Inner Mongolia Normal University. All plants used in this study were artificially cultivated in the laboratory. Quinoa is not listed as a protected or endangered species, and no special permissions or approvals were required for the collection of this plant material. All sampling and experimental procedures involving these plants complied with local regulations, as well as institutional, national, and international guidelines for plant research. Plant species identification was performed by Jiaqi Gu, an author of this study, based on standard morphological characteristics. As the samples were exclusively used for metabolomic analysis, no voucher specimen was deposited.

The plant material used in this study was Qingli 2, a quinoa cultivar with good salt tolerance, which was kindly provided by Gansu Academy of Agricultural Sciences. Two treatment groups were established: a control (CK) group with normal watering and a stress (T) group treated with a 100 mmol/L NaCl: NaHCO₃ mixed solution at a molar ratio of 1:2. Three biological replicates were set up for each treatment. The potting soil consisted of a 2:1 mixture of nutrient soil and organic nutrient soil, which was placed into pots measuring 15 cm in height and 12 cm in diameter. Three quinoa seedlings were planted per pot. Salt stress treatment was initiated 30 days after seedling emergence and lasted for 14 days, after which samples were collected for rhizosphere soil metabolomic analysis. The rhizosphere soil from the three pots of the control group was uniformly mixed and sieved. The sieved soil samples were aliquoted into sterile, enzyme-free centrifuge tubes, with each tube containing no less than 15 g of soil. The same sampling procedure was followed for the treatment group. Six technical replicates for instrumental sequencing analysis were prepared for each group. Following collection, all samples were flash-frozen in liquid nitrogen for 15 min, stored long-term at -80 °C, and transported on dry ice. A 3-gram sample was weighed for metabolite extraction. The freeze-dried sample was placed in a 2mLcentrifuge tube containing 5 mm tungsten beads and pulverized for 1 min at the 65 Hz frequency of the grinder. 1mL pre-cooled methanol-acetonitrile-water mixed extraction solution (v/v/v, 2:2:1) was added, and it was ultrasonicated in an ice bath for 1 h, then left to stand at -2℃ for 1 h, and centrifuged at 4℃, 14000×g for 20 min. The supernatant was collected and concentrated to dryness under vacuum. The metabolome test and analysis were carried out by Shanghai Bioprofile Biotechnology Co., Ltd (http://www.bioprofile.cn/index.aspx).

A metabolomics analysis was performed using UHPLC-Q-Orbitrap-MS (Shimadzu Nexera X2 LC-30AD ultra-high performance liquid chromatograph coupled with Thermo Scientific Q-Exactive Plus mass spectrometer).

For liquid separation, an ACQUITY UPLC^®^ HSS T3 chromatographic column (2.1 × 100 mm, 1.8 μm) was used, with a flow rate of 0.3mL/min. The mobile phase composition: A: 0.1% formic acid aqueous solution; B: 100% acetonitrile. Gradient program: Maintain 0% B from 0 to 2 min; linearly increase to 48% B from 2 to 6 min; increase to 100% B from 6 to 10 min and hold for 2 min; decrease to 0% B from 12 to 12.1 min, and then perform 3 min of column equilibration.

Mass spectrometry data acquisition was performed in both positive and negative electrospray ionization modes. The Heated Electrospray Ionization ion source parameters were as follows: spray voltage 3.8 kV (positive mode)/3.2 kV(negative mode); capillary temperature 320℃; sheath gas flow rate 30arb; auxiliary gas flow rate 5arb; probe heater temperature 350℃; S-Lens RF Level 50. The mass spectrometry scan range was m/z 70–1050, the resolution of the first-order mass spectrometry was 70,000(m/z200), and the resolution of the second-order mass spectrometry was 17,500 (m/z 200). The maximum injection time: 100 MS for the first-order scan and 50 MS for the second-order scan. The MS2 isolation window was 2 m/z, and the normalized collision energy ladder was set to 20, 30, and 40.

Pathway enrichment analysis of differentially abundant metabolites was performed based on the KEGG database. Fisher’s exact test was used for statistical testing, and multiple testing was corrected by False Discovery Rate. Ap < 0.05 was used as the criterion for significant pathway enrichment.

The differential abundance score is a pathway-based method for analyzing metabolic changes, which can capture the overall changes of all metabolites in a certain pathway.

Raw mass spectrometry data were subjected to peak alignment, retention time correction, and peak area extraction using MS-DIAL. Metabolite identification was based on accurate mass numbers (mass tolerance < 10 ppm) and second-order mass spectrometry data (mass tolerance < 0.02 Da), and matched public databases such as HMDB and MassBank, as well as a self-built standard library. Among the extracted ion features, only variables with a non-zero measurement value ratio exceeding 50% in at least one group were retained.

Multivariate data analysis and modeling were performed using R language (version 4.0.3) and related packages. The data were mean-centered. Principal component analysis models and orthogonal partial least squares discriminant analysis models were established respectively. Differentially abundant metabolites were screened using the Variable Importance in Projection (VIP) values obtained from the Orthogonal Partial Least Squares Discriminant Analysis (OPLS-DA) model combined with the p values of two-tailed t-tests (one-way ANOVA was used for multi-group analysis). A VIP > 1.0 and *p* < 0.05 were used as the significance thresholds. The fold change was calculated as the logarithm of the ratio of the average mass spectrometry response values (peak areas) of two types of samples. Cluster analysis of the identified differentially abundant metabolites was performed using the R package.

## Results

### Principal component analysis and orthogonal partial least squares discriminant analysis of the control group and the stress group

**Fig. 1:**
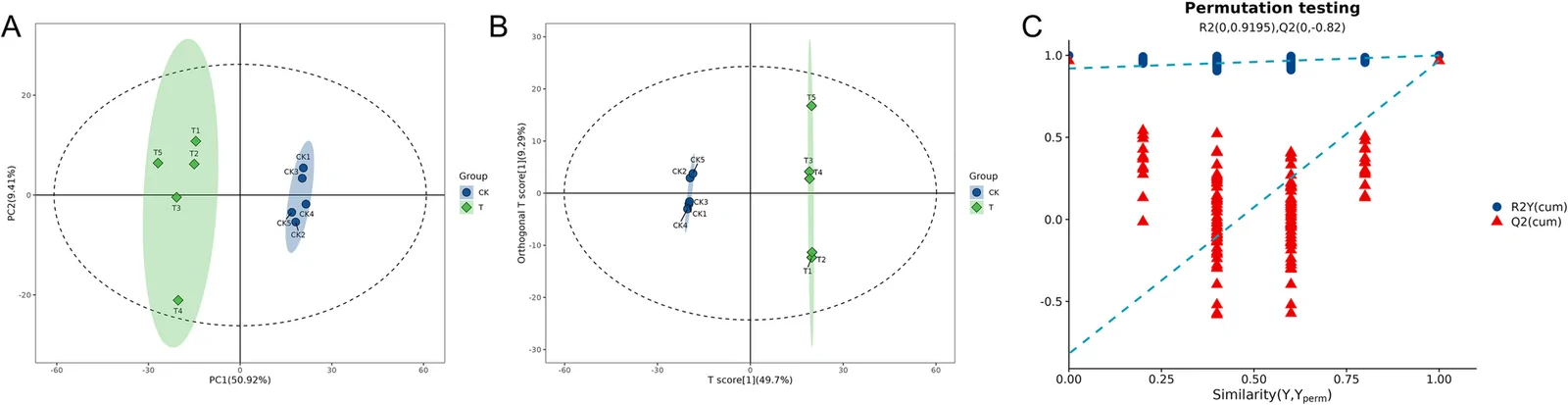
PCA score plot (**A**), OPLS-DA score plot (**B**), and OPLS-DA permutation test plot (**C**) comparing CK and T groups of quinoa rhizosphere soil. **A** and **B** illustrate inter- and intra-group metabolite differences; **C** validates the OPLS-DA model with 200 permutation tests

To explore the effects of salt stress on the rhizosphere soil metabolites of quinoa, non-targeted metabolomics detection technology was used to further analyze the metabolic differences in the experimental samples. Before analyzing the differences in metabolites among groups, principal component analysis (PCA) and OPLS-DA were performed to observe the variability between different groups and within samples of each group. The results are shown in Fig. 1. The PCA result in Fig. 1A shows that the samples of CK and T showed a certain degree of separation trend. The contribution rates of the first principal component (PC1) and the second principal component (PC2) were 50.92% and 9.41% respectively, cumulatively explaining 60.33% of the total data variability. This indicates that salt stress has changed the composition of the rhizosphere soil metabolites of quinoa, resulting in a certain degree of differentiation between the two groups of samples in the principal component space. In the OPLS-DA result in Fig. 1B, the predictive component (t₁) captures the variation between groups, while the orthogonal component (t₁⊥) captures variation unrelated to group separation. To further validate the reliability of the OPLS-DA model, a permutation test (200 permutations) was performed (Fig. 1C). The results showed R² = 0.9195 and Q² = -0.82, with the Q² regression line intercepting the y-axis at a negative value, indicating that the original model was not overfitted and the observed separation is statistically robust. Combining the results of PCA and OPLS-DA analysis, it shows that salt stress reshapes the rhizosphere soil metabolic network.

### Quality control (QC)

Quality control samples were constructed by mixing sample extracts in equal proportions to evaluate the stability of the experimental procedure [16].

**Fig. 2:**
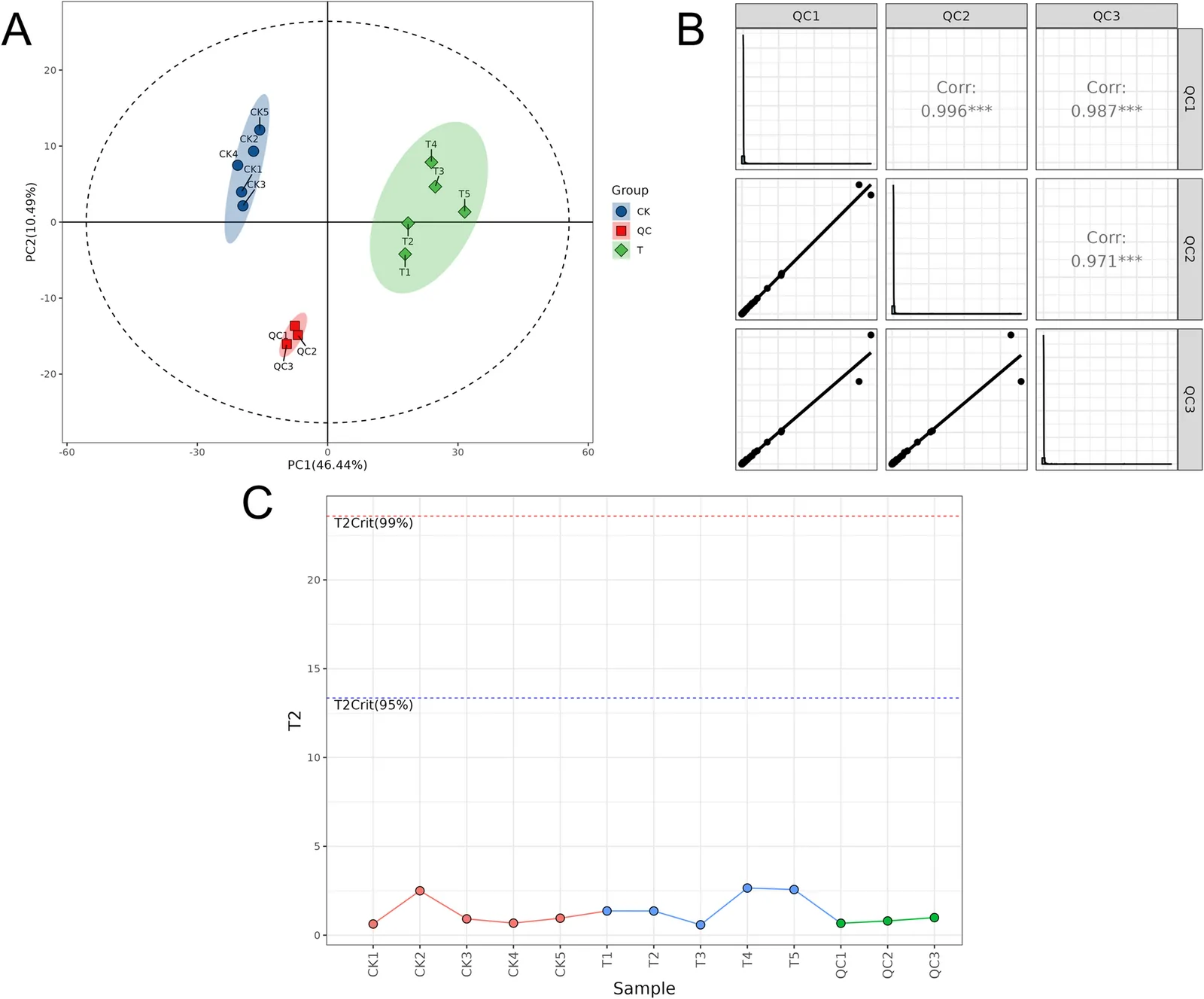
Quality control analysis chart of quinoa soil samples. **A** PCA plot (merged modes); **B** QC correlation plot (merged modes); **C** Hotelling’s T2 test plot (merged modes)

After data standardization of the peaks obtained from all experimental samples and QC samples, PCA analysis was performed, as shown in the figure below. The PCA analysis results of the differential metabolites in the rhizosphere of quinoa under different treatments are shown in Fig. 2A. As can be seen from the figure, the QC samples are all concentrated in the off-center position, with good stability and reproducibility, and can be used for metabolomics analysis of quinoa samples. The contribution rates of the three principal components in the PCA score plot are 46.44%, 10.49%, and 7.89% respectively. The contribution rates of the three principal components exceed 60%. At the same time, the soil metabolites under different treatments converge significantly within the group, and the distance between samples in different groups is far, and the clustering effect is significant in the spatial distribution. Among them, the salt stress group T is located on the right side of the first principal component axis, while the control group CK and the quality control group QC are located on the left side, and the overall spatial distance from other varieties is far. Pearson correlation analysis was performed on the QC samples. Generally, a correlation coefficient greater than 0.9 indicates a good correlation. The results of the quality control correlation matrix shown in Fig. 2B indicate that the three QC indicators analyzed are highly consistent and the change trends are exactly the same. Hotelling’s T2 test is used to test the samples through multivariate modeling, defining 95% or 99% confidence intervals, which can be used for the diagnosis of outlier samples. As shown in Fig. 2C, the same group is used for the same color. The T2 values of all sample points (CK1-5, T1-5, QC1-3) are close to 0, and all are far below the blue 95% control line and even farther below the red 99% control line. This indicates that the multivariate statistics of all samples are within the expected range, and no outliers or outlier points appear. The T2 values of the control group (CK, red) and the treatment group (T, blue) are at the same low level and there is no significant difference. This indicates that from the overall model, the treatment group does not introduce strong abnormal fluctuations. It shows that the detection experiment has good repeatability, very good system stability, controllable and reliable data quality, providing a solid data quality basis for subsequent formal data analysis.

### Screening and analysis of differential metabolites

**Fig. 3:**
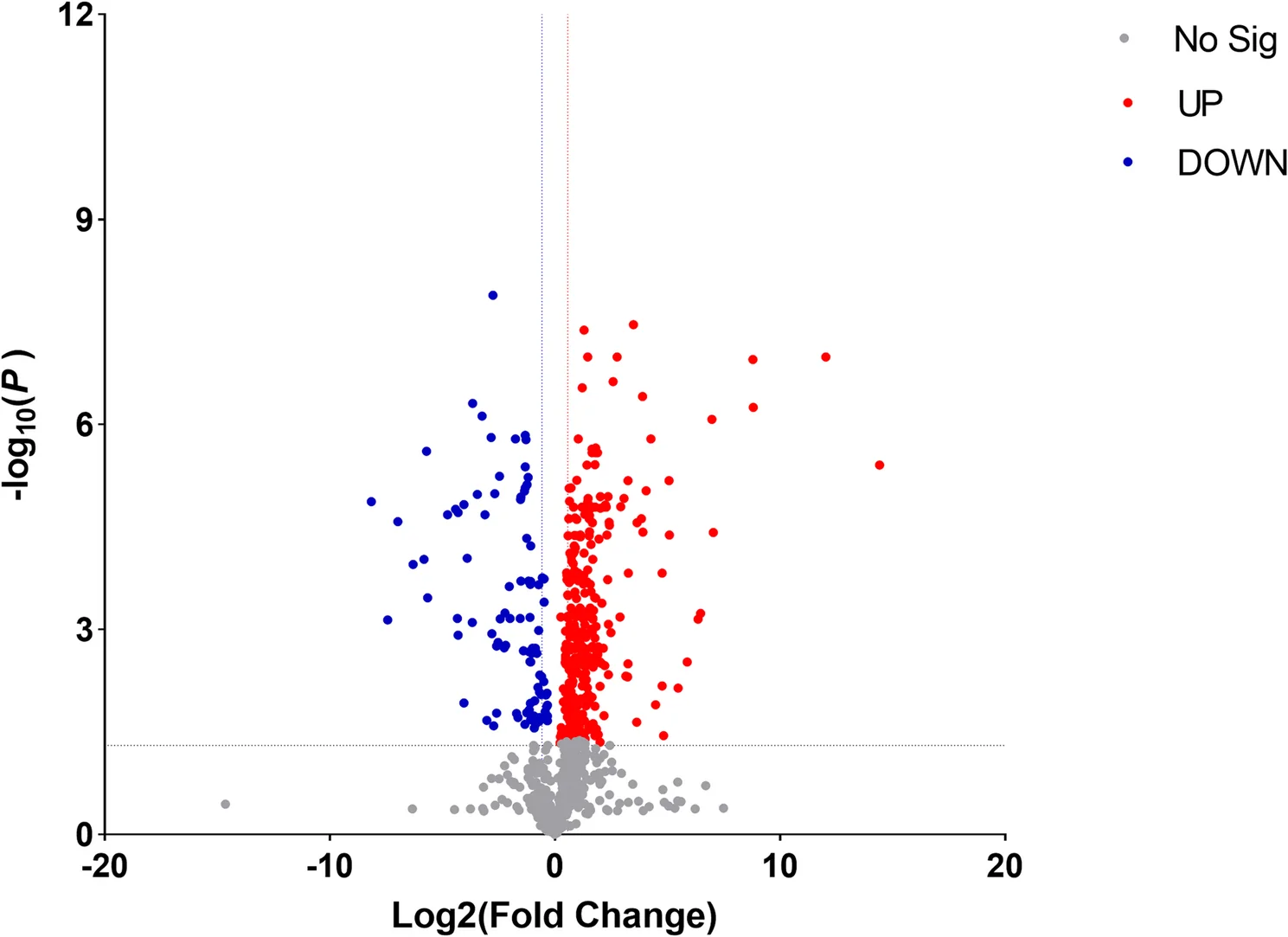
Volcano plot (T vs. CK). Blue indicates downregulation, red indicates upregulation

After OPLS-DA analysis, metabolites with Fold Change (FC) > = 1.5 or FC < = 1/1.5 and P.value < 0.05 were used as criteria to screen for differential metabolites. In this discovery‑oriented untargeted metabolomics study, we deliberately did not apply multiple testing correction (such as FDR) to the p‑values at the individual metabolite level, in order to avoid over‑correction that could lead to the loss of potential candidate metabolites. Among the 856 metabolites detected, a total of 439 differential metabolites were screened out in T vs. CK (Fig. 3 ), including 344 up-regulated metabolites and 95 down-regulated metabolites. Under salt stress conditions, the content of up-regulated metabolites was much higher than that of down-regulated metabolites, indicating that salt stress had a certain impact on the metabolites in the rhizosphere soil of quinoa.

Volcano plot of differential expression shows metabolic changes; the x-axis is log₂ (fold change), y-axis is -log₁₀(p), and colors distinguish up-regulated, down-regulated and insignificant metabolites.

**Fig. 4:**
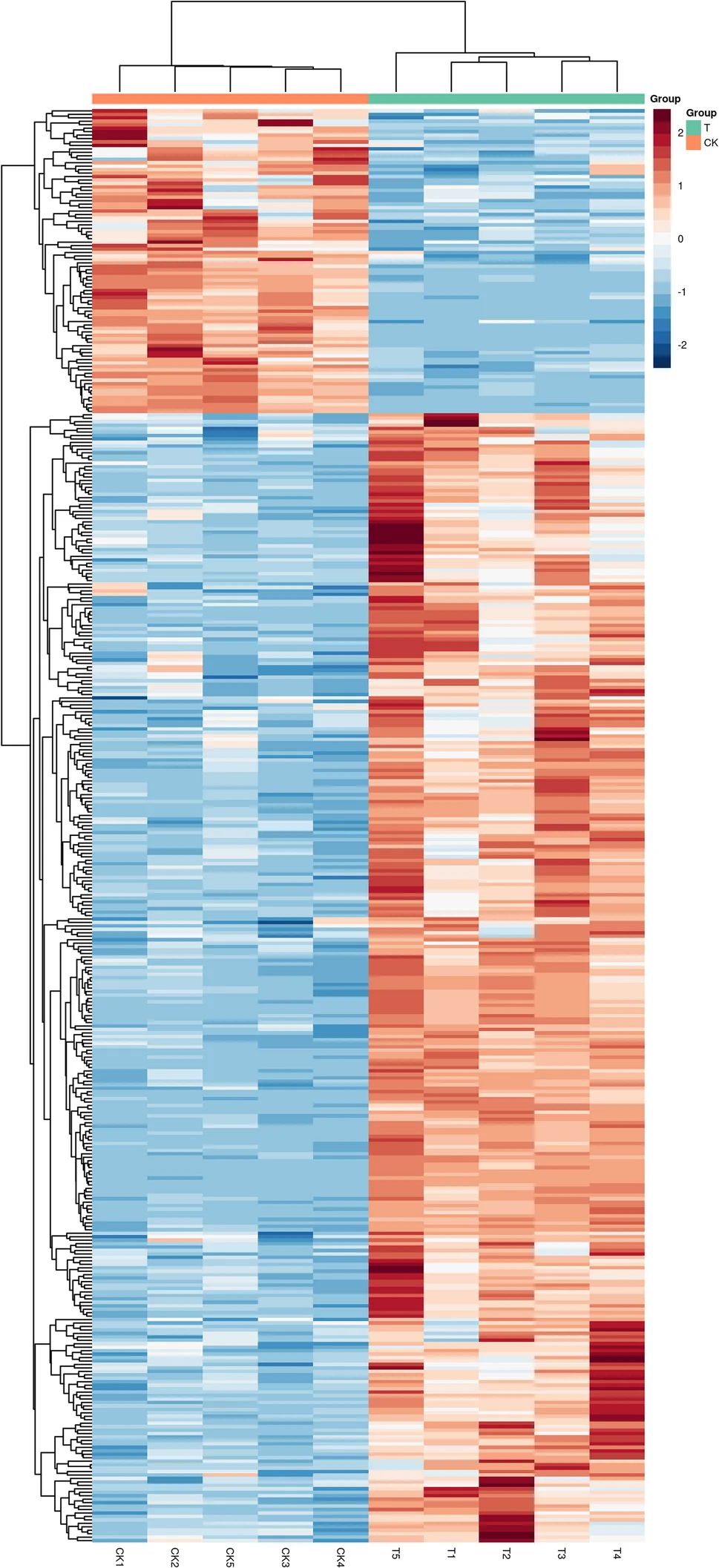
Comparative group T vs. CK differential metabolites hierarchical clustering result diagram. Clustered heatmap of differential abundance shows feature abundance distribution in different groups; red indicates high abundance, blue low abundance, and the dendrogram reflects feature relatedness

The relative abundance distribution of secondary metabolites in quinoa rhizosphere soil under salt stress is shown in Fig. 4. The standardized color gradient was used to reflect the expression changes of metabolites. The heatmap was clearly divided into two major clusters, corresponding to the control group and the stress group respectively, further verifying the results of PCA and OPLS-DA analysis. There was obvious clustering within the group and good repeatability. In addition, by observing the heatmap, it was found that certain specific metabolites were significantly enriched, and these metabolites might have a close relationship with salt stress, providing important clues and directions for subsequent screening of differential metabolites and functional research.

### Classification of differential metabolites

**Fig. 5:**
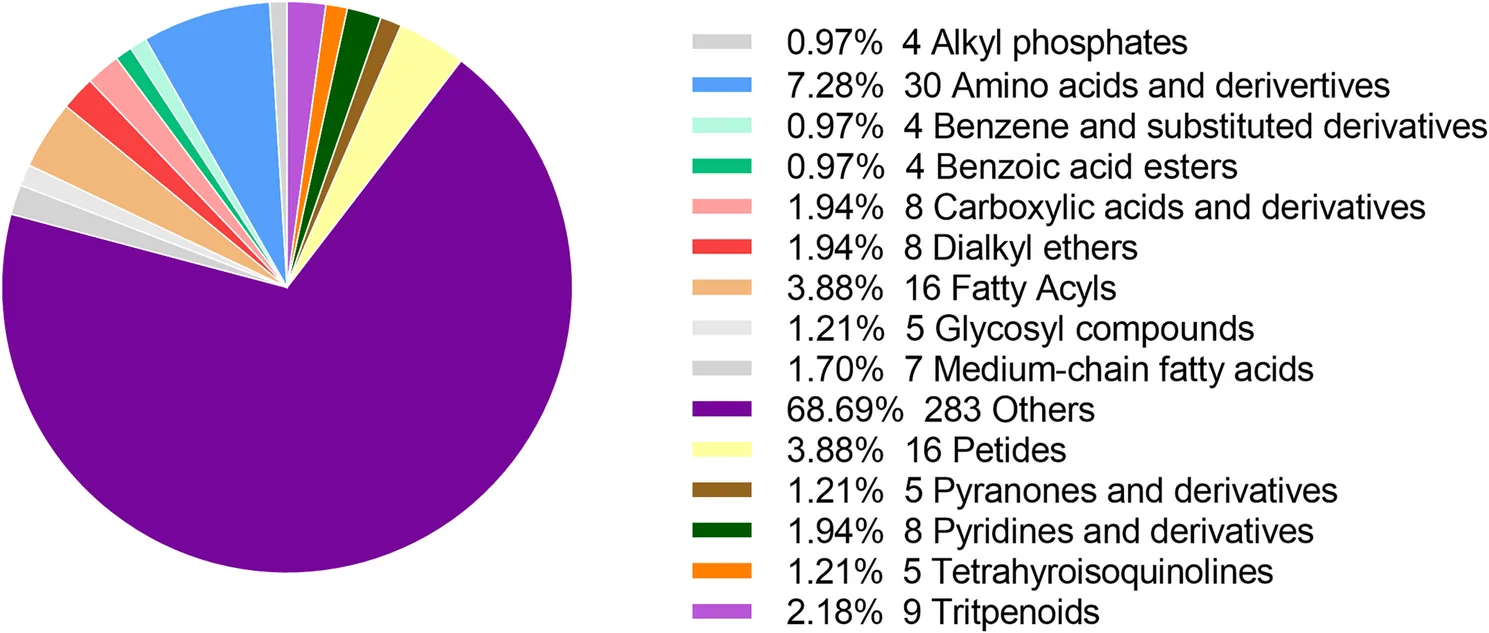
Classification ring chart of differential metabolites in T vs. CK comparison group. Colors indicate categories, areas indicate counts, and pie chart shows proportions

In this study, 856 metabolites were detected in the control group and the stress group using UHPLC-Q- Orbitrap -MS technology, including 283 other metabolites (68.69%), 30 amino acids and their derivatives (7.28%), 16 fatty acyls (3.88%), 16 polypeptides (3.88%), 9 triterpenoids (2.18%), 8 carboxylic acids and their derivatives (1.94%), 8 dialkyl ethers (1.94%), 8 pyridines and their derivatives (1.94%), 7 medium-chain fatty acids (1.7%), 5 glycosyl compounds (1.21%), 5 pyranones and their derivatives (1.21%), 5 tetrahydroisoquinolines (1.21%), 4 alkyl phosphates (0.97%), 4 benzenes and their substituted derivatives (0.97%), and 4 benzoates (0.97%), as shown in Fig. 5. Upon salt stress treatment, the top 30 differential metabolites in quinoa rhizosphere soil ranked by VIP values are listed in Table 1. These metabolites include: organic acids and their derivatives (9 types), organic oxygen compounds (9 types), lipids and lipid-like molecules (5 types), organoheterocyclic compounds (2 types), organic nitrogen compounds (2 types), benzenoids (1 type), phenylpropanoids and polyketides (1 type), and nucleosides, nucleotides, and analogues (1 type). Under salt stress, the rhizosphere metabolome exhibited significant accumulation of multiple compound classes associated with stress defense. Lipids and lipid-like molecules, which may modulate membrane properties and transduce stress signals, were prominently increased. Small-molecule organic acids, sugars, and other compatible solutes, known for their roles in osmotic balance and protection of cellular structures, were also enriched. Additionally, the phenylpropanoid pathway, involved in antioxidant defense and cell wall reinforcement, was activated. Nucleotides and nitrogen-containing compounds, linked to energy metabolism and signal transduction, showed altered abundances. Collectively, these coordinated metabolic shifts in the rhizosphere are consistent with a systemic adaptive response, although their precise source allocation among plant roots, microbial communities, and soil physicochemical processes cannot be resolved from the present data. 

**Table 1:** Key differential metabolites in T and CK groups

Metabolite name	Superclass	Significant
Betaine aldehyde	Organic nitrogen compounds	UP
Clovamide	Organic acids and derivatives	DOWN
Maltose	Organic oxygen compounds	DOWN
Carnitine	Organic nitrogen compounds	UP
Allantoic acid	Organic acids and derivatives	DOWN
Glycerophosphocholine	Lipids and lipid-like molecules	UP
Jasmonic acid	Organic acids and derivatives	UP
Gentiobiose	Organic oxygen compounds	DOWN
Docosanedioic acid	Lipids and lipid-like molecules	UP
beta-carboline	Organoheterocyclic compounds	UP
Ectoine	Organic acids and derivatives	UP
Melibiose	Organic oxygen compounds	UP
Suberic acid	Lipids and lipid-like molecules	UP
Lactulose	Organic oxygen compounds	DOWN
Raffinose	Organic oxygen compounds	DOWN
Kynurenic acid	Organoheterocyclic compounds	UP
Inosine	Nucleosides, nucleotides, and analogues	UP
Mannitol	Organic oxygen compounds	DOWN
Gallic acid methyl ester	Benzenoids	UP
1-Aminocyclopropane-1-carboxylic acid	Organic acids and derivatives	UP
Proline	Organic acids and derivatives	UP
Eicosanedioic acid	Lipids and lipid-like molecules	UP
Sucrose	Organic oxygen compounds	DOWN
D-Sorbitol	Organic oxygen compounds	UP
Formononetin	Organic acids and derivatives	UP
LPC (18:1)	Lipids and lipid-like molecules	UP
Tyrosine	Organic acids and derivatives	UP
Phenylalanine	Organic acids and derivatives	UP
4-Coumaroylcholine	Phenylpropanoids and polyketides	UP
Melezitose	Organic oxygen compounds	UP

### Differential metabolite Kyoto Encyclopedia of Genes and Genomes (KEGG) enrichment analysis

**Fig. 6:**
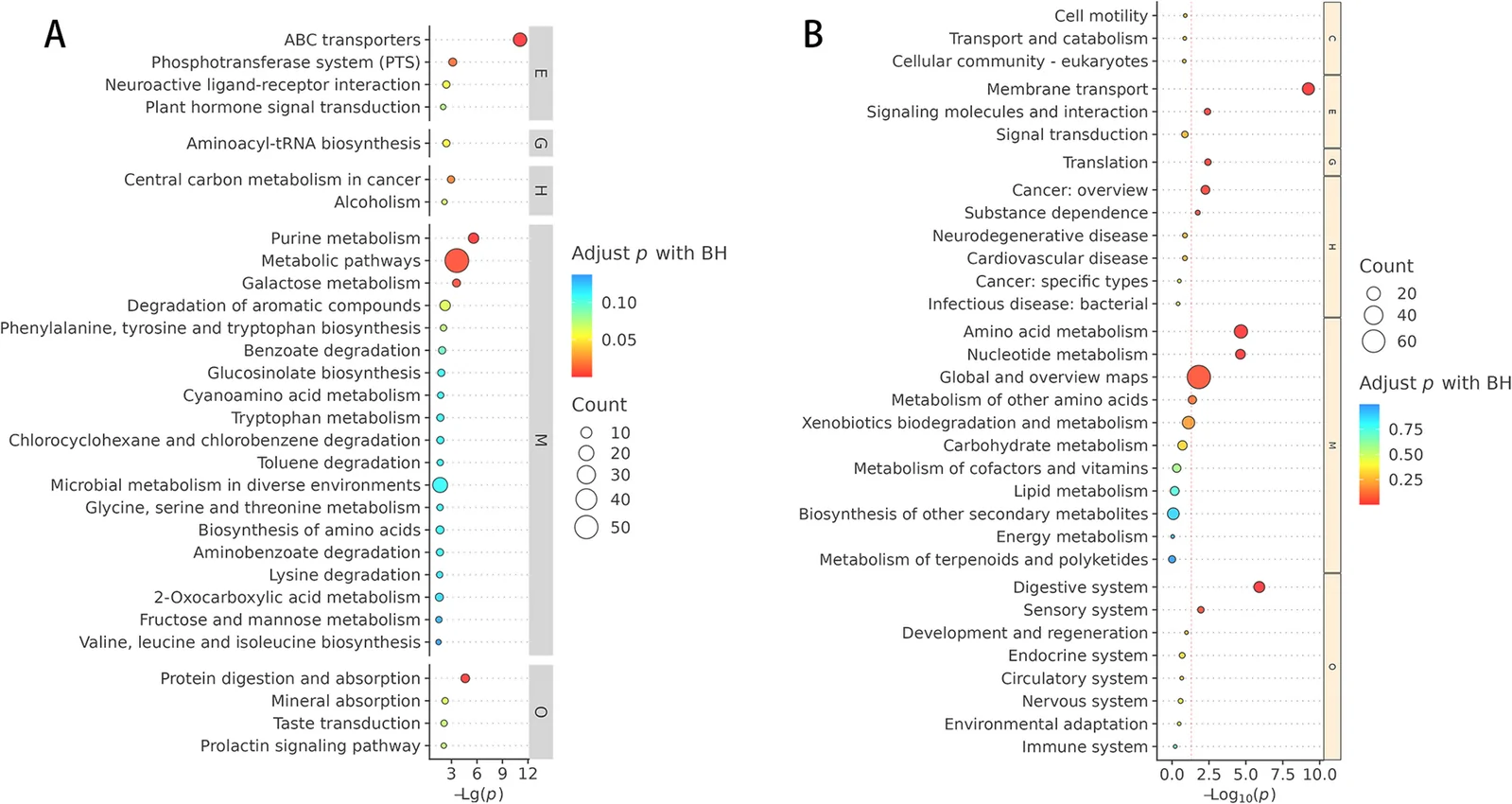
KEGG enrichment map of differential metabolites in the CK group compared to the T group. **A** Enrichment bubble plot of the top 30 significantly enriched specific pathways belonging to KEGG Level‑1 classifications; **B** Enrichment statistical bubble plot of KEGG Level‑2 pathway subcategories. Level 1 pathway classification: Metabolism (M), Genetic Information Processing (G), Environmental Information Processing (E), Cellular Processes (C), Organismal Systems (O), Human Diseases (H), Drug Development (D) Some KEGG pathway labels shown in the figure are generic ontology terms derived from the KEGG database and do not represent biologically meaningful functional categories for rhizosphere metabolism in this study. Their appearance reflects the broad annotation coverage of the KEGG database rather than specific physiological processes in the plant-soil system

According to KEGG, pathway enrichment analysis was performed. As shown in, the analysis was conducted for the top 30 most significant metabolic pathways. The vertical axis in the figure lists the top 30 significant pathways, with the significance decreasing from top to bottom within the group; the horizontal axis -log10(p) value reflects statistical significance. Most data points are blue and small in size, indicating that after salt stress treatment, the basic metabolic state of the soil microorganisms in the quinoa root system is relatively stable, and the changes in the intracellular metabolic network are relatively mild. In the enrichment results (Fig. 6A), in the first layer, under salt stress, pathways related to antioxidant, defense, and energy metabolism were significantly enriched, including phenylalanine metabolism, amino acid biosynthesis, and ABC transporters. Combining the differential metabolite categories in these pathways, KEGG enrichment analysis shows that a synergistic network composed of the three major pathways of phenylalanine metabolism, amino acid biosynthesis, and ABC transporters jointly dominates. Phenylalanine metabolism performs the functions of reactive oxygen scavenging and cell wall strengthening by producing antioxidants such as flavonoids and lignin precursors respectively; the amino acid biosynthesis pathway achieves osmotic regulation by accumulating a large amount of compatible solutes such as proline and provides precursors for stress-resistant metabolism; Metabolites associated with the ABC transporter pathway were among those significantly enriched, suggesting a potential role in ion and metabolite transport under salt stress. These three major pathways were among the most significantly enriched pathways, suggesting a coordinated metabolic response involving defense-related compound synthesis, osmotic protection, and transport regulation within the rhizosphere system.

As can be seen from Fig. 6B, in the second-layer enriched pathways, the number of differential metabolites in the global and overview maps is the largest and very significant. The significance of amino acid metabolism, nucleotide metabolism, and membrane transport is high, and the number of metabolites in the metabolic pathways is also relatively large. The pathways belonging to metabolism in the KEGG Level 1 classification are the most, followed by biological systems. KEGG enrichment analysis revealed coordinated changes across multiple metabolic pathways, suggesting that the rhizosphere metabolic network undergoes systematic reprogramming in response to salt stress. Under this framework, amino acid and nucleotide metabolism respectively provide osmoprotective substances, energy, and signaling molecules for the defense response; the membrane transport system maintains homeostasis by precisely regulating ion and metabolite transport; and the resource mobilization related to the digestive system provides backup support for this series of energy-consuming processes, jointly constituting a complete salt tolerance regulation system from environmental perception to physiological function execution.

### Analysis of the overall changes in the metabolic pathways of differential metabolites

**Fig. 7:**
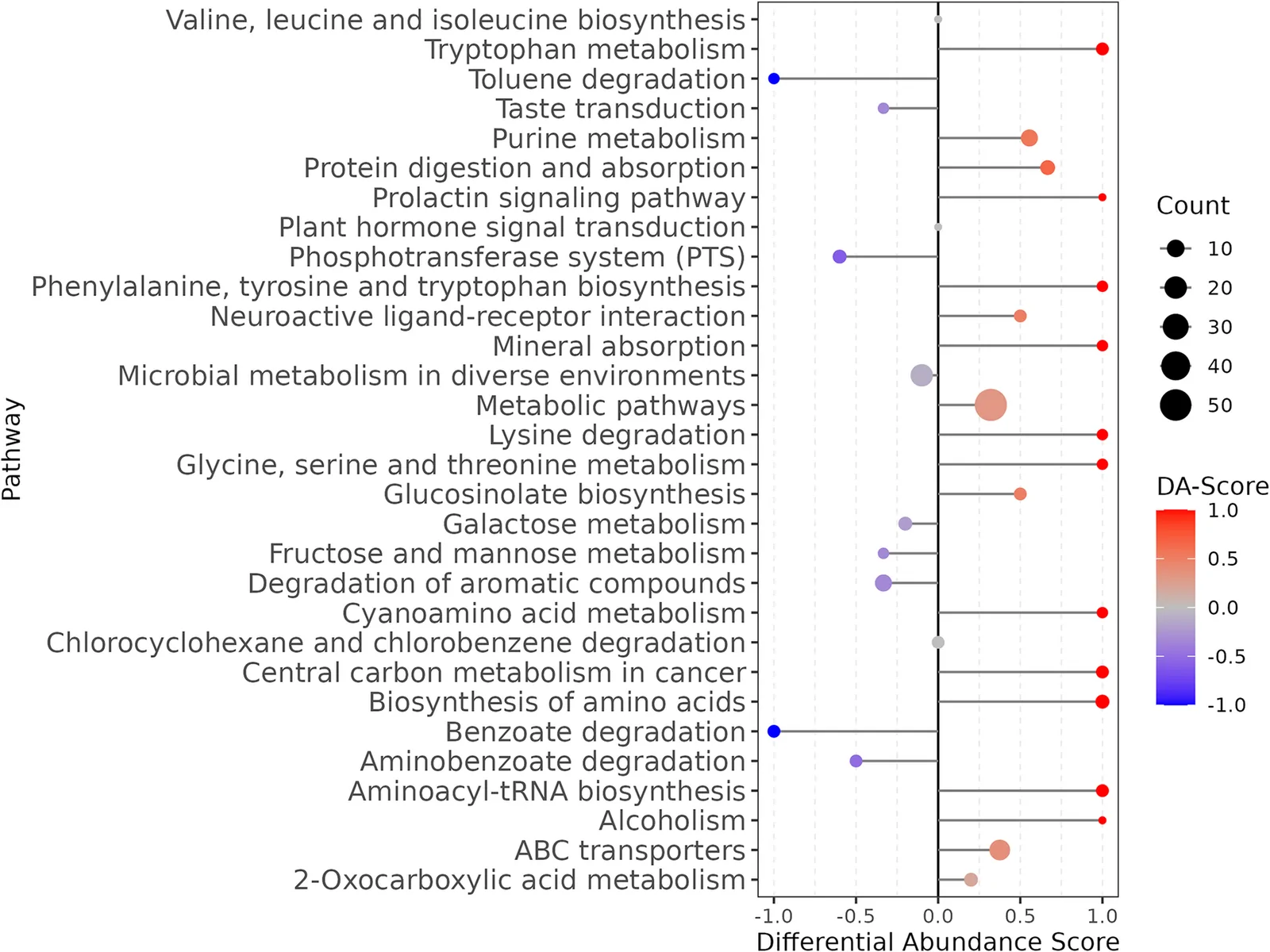
Differential abundance score plot for T vs. CK comparison. The DA score of 1/-1 indicates the up/downregulation of metabolic pathways; segment length shows strength; larger dots indicate more differential metabolites; color represents the *p*-value

The Differential Analysis (DA) Scores of most metabolic pathways are concentrated in the positive value range, and the DA Scores of most pathways are significantly deviated from zero, which reflects that under salt stress, the differential metabolites involved in these pathways in the rhizosphere are mainly up‑regulated, reflecting the extensive activation of rhizosphere metabolic activities, which result from a coordinated plant‑microbe symbiotic response. In Fig. 7, the closer the DA score of metabolic pathways such as tryptophan metabolism, lysine degradation, glycine, serine, threonine metabolism is to 1, the more the overall expression tends to be up-regulated, suggesting their potential importance in the rhizosphere stress response. The DA scores of toluene degradation and benzoic acid degradation are − 1, and the abundances of all metabolites in the pathway decrease, and their metabolic activities are significantly inhibited under salt stress. The metabolic pathway has the largest number of metabolites. Salt stress tends to activate the activities of key metabolic pathways, possibly due to enhanced enzyme activity, degradation of amino acid metabolites, or a shift in the overall metabolic balance towards catabolism. These changes in metabolic pathways reveal the overall characteristics and key changes in soil metabolic reprogramming under salt stress, providing an important basis for in-depth understanding of the metabolic adaptation mechanism of plants under adverse conditions.

### Network diagram of enriched pathways

**Fig. 8:**
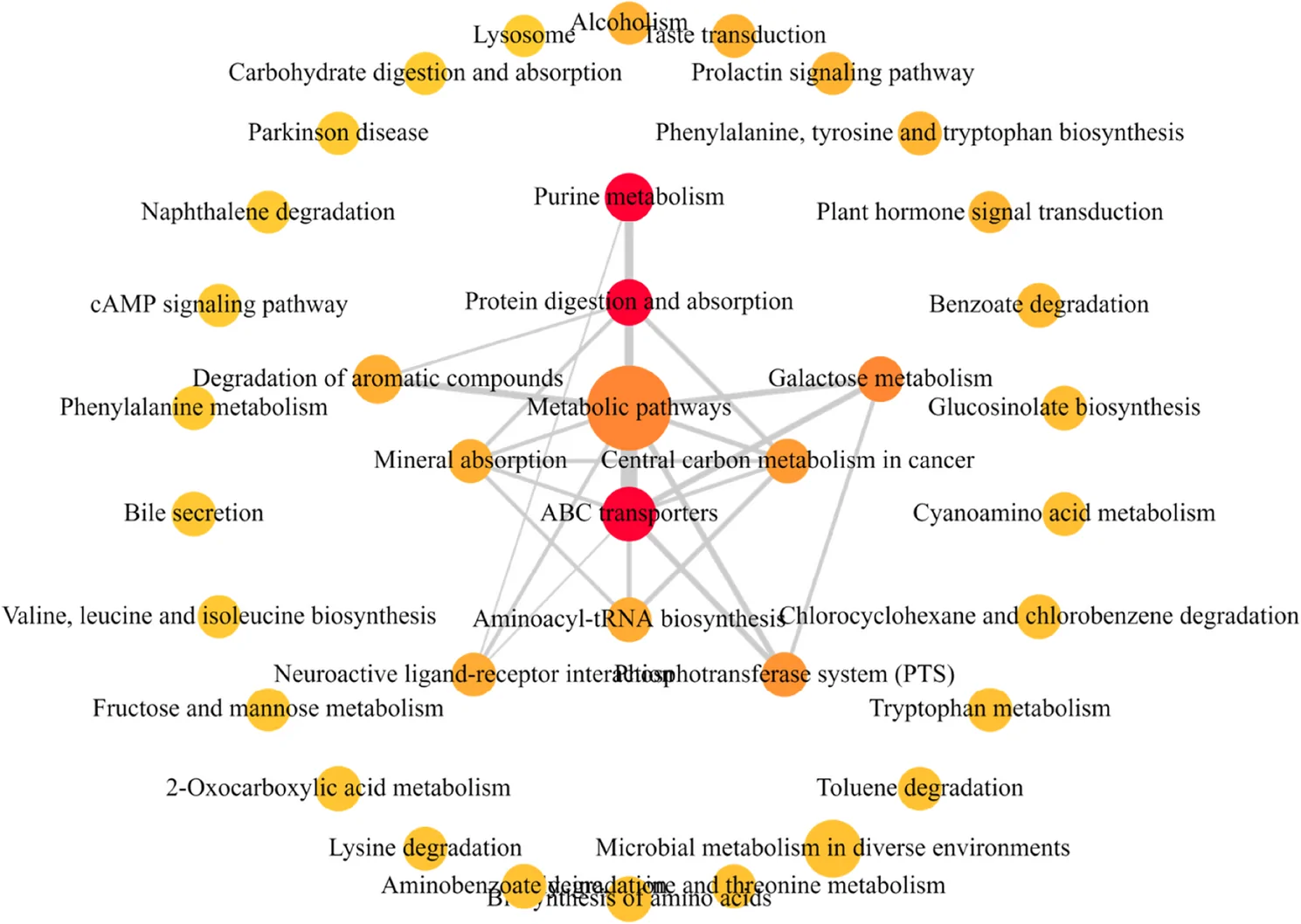
Network diagram of enriched pathways in the comparison group T. vs. CK. This network illustrates functional interactions among pathways. Nodes represent pathways, colored by enrichment significance (redder = more significant) and sized by metabolite count. Edges indicate shared differential metabolites, with thickness representing the quantity shared

The KEGG enrichment pathway network is shown in Fig. 8, pathways such as the ABC transport pathway, purine metabolism, metabolic pathways, and galactose metabolism were significantly enriched, with a large number of metabolites. Purine metabolism and protein digestion and absorption, metabolic pathways and protein digestion and absorption, and metabolic pathways and the ABC transport pathway contained common differential metabolites, and there were many metabolites. Under salt stress, soil microorganisms activate key metabolic pathways and form a co-regulation network through metabolite cross-nodes to achieve the overall reconstruction of the metabolic network, thereby maintaining their survival and functional stability under adverse conditions.

The KEGG enrichment network revealed significant co-enrichment of pathways including ABC transporters, protein digestion and absorption, purine metabolism, and galactose metabolism, with numerous shared differential metabolites serving as cross-pathway nodes. At the same time, this process also enhances the activation and supply of mineral nutrients such as phosphorus and iron in the soil, alleviating the ionic toxicity and nutrient stress of plants [17]; Meanwhile, the active processes of protein digestion and absorption and purine metabolism drive the conversion of the rhizosphere organic nitrogen pool into a form easily absorbed by plants, and especially synthesize the plant growth hormone (with tryptophan as a precursor, thereby stimulating the development of plant roots and expanding the absorption area to cope with salt stress [18]; And a large number of differential metabolites such as amino acids and nucleosides shared between these pathways act as cross-nodes to achieve the overall reconstruction and functional stability of the metabolic network, ensuring that a metabolically highly connected and robust rhizosphere microbial community can continuously provide nutrients and signal support for plants, systematically strengthening the salt tolerance of plants [19].

## Discussion

Metabolomics can effectively reveal the response mechanism of plants by monitoring the changes in metabolites after stress, which is a good way to study plant stress resistance [20]. In this study, based on UHPLC-Q-Orbitrap-MS technology, the metabolites in the rhizosphere soil of quinoa under salt stress were analyzed. The results showed that there were more than 400 differential metabolites, and most of the differential metabolites in each treatment group were up-regulated. These up-regulated metabolites were mainly distributed across superclasses such as lipids and lipid-like molecules, organic acids and derivatives, organooxygen compounds, organoheterocyclic compounds, and benzenoids, which collectively constituted the primary responding components. Notably, differential metabolites included ectoine, proline, and 1-aminocyclopropane-1-carboxylic acid, mannitol and D-sorbitol, glycerophosphocholine and docosanedioic acid, beta-carboline and kynurenic acid, as well as gallic acid methyl ester and 4-coumaroylcholine.

A total of more than 400 differential metabolites were identified in this study, and most differential metabolites were significantly upregulated across all treatment groups. These upregulated metabolites were mainly lipids and lipid-like molecules, organic acids and their derivatives, organooxygen compounds, organoheterocyclic compounds and benzenoids, which constituted the core metabolic components of quinoa in response to salt stress. Such metabolic patterns are widespread among halophytes. Duan et al. [21]conducted metabolomic research on Salicornia europaea and verified that salt stress triggers massive accumulation of differential metabolites, among which lipids and organic acids act as key functional substances for salt tolerance. Studies focusing on abiotic stress in quinoa have yielded consistent results. Zhu et al. [22] detected prominent enrichment of amino acids, lipids and organic acids in quinoa leaves under drought stress, further confirming that lipids and organic acids serve as central responsive metabolites when quinoa copes with various abiotic stresses.

The lactose metabolism pathway was the most significantly remodeled pathway observed in the present research. This finding is consistent with the results of the changes in metabolites in the glycolysis and pentose phosphate pathways observed by Zhang et al. [23]in the leaves of Cyclocarya paliurus under salt stress. Integrated transcriptomic and metabolomic analyses of salt-tolerant rice roots conducted by Yang et al. [24] also revealed that the galactose metabolism pathway actively responds to salt stress, accompanied by massive accumulation of saccharides such as lactose and sucrose. This further verifies that metabolic reprogramming of carbohydrates is a conserved physiological strategy adopted by plants to resist salinity. In this study, the lactose metabolism pathway was one of the most significantly changed pathways. As the main energy source for various life activities of plants, sugars provide energy for plant growth, metabolism and reproduction, and also participate in the process of regulating plant adaptation to environmental changes [25]. As primary metabolites in plants, non-structural carbohydrates are crucial for plant growth, not only maintaining energy supply but also participating in structure construction [26]. In this experiment, the lactose metabolism pathway was among the most significantly altered pathways in the rhizosphere under salt stress, which may reflect shifts in carbon allocation and osmotic adjustment within the rhizosphere system.

From the perspective of the main differential metabolites, the differential metabolites in the rhizosphere soil of quinoa were distributed across multiple superclasses, including organic acids and their derivatives, organooxygen compounds, lipids and lipid-like molecules, organoheterocyclic compounds, benzenoids, and phenylpropanoids.

Under salt stress, the amino acid metabolism in the rhizosphere, involving both plant roots and their associated microbes, is enhanced, indicating a coordinated symbiotic metabolic response rather than a solely microbial process. A study by Ahmad et al. demonstrated that proline in tomatoes can regulate cellular metabolic activities and maintain cell membrane stability under salt stress [27]. The upregulation of amino acid metabolites observed in this study is consistent with the known osmotic protective functions of compatible solutes. Abscisic acid, a key stress hormone, has also been reported to enhance plant tolerance to osmotic stress [28], and changes in its metabolic pathways may be embedded within broader metabolic regulatory networks. KEGG enrichment analysis revealed significant differences in core metabolic pathways induced by salt stress, with amino acid metabolism and nucleotide metabolism pathways being notably enriched. This reflects the increased demand for energy metabolism and the synthesis of osmoregulatory substances in plants under salt stress [29].

In this study, through cluster analysis, it was found that salt stress promoted the significant accumulation of differential metabolites such as organic acids and their derivatives, organooxygen compounds, benzenoids and phenylpropanoids, as well as lipids and lipid-like molecules. These accumulated metabolites are known from previous studies to contribute to osmotic adjustment and cellular protection. Their increased abundance in the rhizosphere under salt stress is consistent with a potential role in salt adaptation. The research by Yu et al. shows that [30], under salt stress, Suaeda salsa can accumulate organic acids, soluble sugars, lipid metabolites, unsaturated fatty acids and sucrose, which not only help the plants cope with osmotic stress but also improve their nutritional value.

Under salt stress, the adaptive responses of plants involve a series of complex metabolic reprogramming processes, in which various small-molecule metabolites act synergistically to form multi-dimensional defense strategies. The accumulation of organic acids not only directly participates in osmotic adjustment to maintain cellular water balance but also alleviates ion toxicity by promoting vacuolar sequestration of Na⁺ and chelating metal ions [31]. Their synthesis and metabolic processes simultaneously play a crucial role in intracellular pH regulation. Compatible solutes such as sugars and sugar alcohols, characterized by their electrically neutral properties, can safely accumulate in the cytoplasm, effectively reducing water potential, stabilizing macromolecular structures and membrane integrity, and often synergizing with the antioxidant system to mitigate oxidative damage. The phenylpropanoid metabolic pathway demonstrates defensive diversity. Its products, such as flavonoids and phenolic acids, exhibit strong antioxidant activity. At the same time, this pathway strengthens cell walls through enhanced lignification, restricts Na⁺ influx, and improves mechanical support [32]. Intermediate products like salicylic acid can also mediate systemic signaling and cross-tolerance. Nucleosides and nucleotides play a dual role in maintaining energy homeostasis and signal transduction [33]. Nitrogen-containing compounds and heterocyclic metabolites, such as jasmonic acid, pipecolic acid, betaine aldehyde, and proline, play key roles in coordinating signaling networks, providing direct antimicrobial protection, and offering osmotic protection. Furthermore, the response of lipids and lipid-like molecules is particularly significant [34]. Nitrogen-containing compounds and heterocyclic metabolites, such as jasmonic acid, pipecolic acid, betaine, and proline, play key roles in coordinating signaling networks, providing direct antimicrobial protection, and offering osmotic protection. Furthermore, the response of lipids and lipid-like molecules is particularly significant [35]. These lipid-related metabolites may contribute to maintaining membrane integrity, providing energy, and serving as signaling molecules that potentially coordinate adaptive responses. However, their precise roles and origins within the plant-soil system require further investigation through complementary approaches. In summary, plant stress resistance under salt stress relies on the integration and interaction of multiple metabolic pathways, forming a comprehensive metabolic network encompassing osmotic adjustment, ion homeostasis, redox balance, and signal regulation. This provides an important theoretical basis and metabolic engineering targets for the genetic improvement and physiological regulation of crop salt tolerance.

## Conclusions

This study used a non-targeted metabolomics method based on liquid chromatography-mass spectrometry to systematically analyze the dynamic changes in metabolites of the rhizosphere of quinoa under salt stress. A total of 856 metabolites were identified, distributed in 15 major categories, among which metabolites in other categories, amino acids and their derivatives, fatty acyls and other metabolic components accounted for 79.85%. Multiple statistics and KEGG pathway enrichment showed that the overall differences in the metabolome were limited. They were mainly concentrated in basic metabolic pathways and the change range was limited. Significant fluctuations occurred in key functional pathways and the levels of functional components. Pathways such as ABC transporter pathway, protein digestion and absorption, purine metabolism, metabolic pathway, and galactose metabolism were significantly enriched, suggesting a coordinated reprogramming environmental stress towards enhanced energy metabolism, nutrient turnover, and transmembrane transport efficiency and functional stability. The benzoate/toluene degradation pathway was significantly up-regulated. These pathways related to the degradation of environmental pollutants showed that altered metabolic capacity in the rhizosphere under stress. Our metabolomic data reveal significant changes in rhizosphere metabolite pools of quinoa under combined saline-alkali stress, with obvious enrichment of osmoprotectants and defensive secondary metabolites. Such metabolomic shifts provide a molecular-level basis for understanding quinoa’s adaptation to saline-alkali conditions. This study offers a theoretical foundation for further research into rhizosphere metabolic regulation and may inform strategies for improving crop performance in saline-alkali lands.

## Data Availability

The datasets used and analysed during the current study are available from the corresponding author on reasonable request.

## Abbreviations

PCA: Principal Component Analysis
KEGG: Kyoto Encyclopedia of Genes and Genomes
DA: Differential Analysis
UHPLC-Q-Orbitrap-MS: Ultra-high performance liquid chromatography coupled with quadrupole-Orbitrap mass spectrometry
OPLS-DA: Orthogonal Partial Least Squares Discriminant Analysis
QC: Quality Control
FC: Fold Change
VIP: Variable Importance in Projection
CK: Control group
T: Treatment (Salt-stress) group
ABC transporters: ATP-Binding Cassette transporters
OsDRAP1: Oryza sativa DREB-related AP2 1
